# Oxygen restriction increases the infective potential of *Listeria monocytogenes in vitro *in Caco-2 cells and *in vivo *in guinea pigs

**DOI:** 10.1186/1471-2180-7-55

**Published:** 2007-06-14

**Authors:** Jens Bo Andersen, Bent B Roldgaard, Bjarke Bak Christensen, Tine Rask Licht

**Affiliations:** 1 National Food Institute, Technical University of Denmark, Mørkhøj Bygade 19, DK-2860 Søborg, Denmark

## Abstract

**Background:**

Listeria monocytogenes has been implicated in several food borne outbreaks as well as sporadic cases of disease. Increased understanding of the biology of this organism is important in the prevention of food borne listeriosis.

The infectivity of *Listeria monocytogenes *ScottA, cultivated with and without oxygen restriction, was compared in *vitro *and *in vivo*. Fluorescent protein labels were applied to allow certain identification of *Listeria *cells from untagged bacteria in *in vivo *samples, and to distinguish between cells grown under different conditions in mixed infection experiments.

**Results:**

Infection of Caco-2 cells revealed that *Listeria *cultivated under oxygen-restricted conditions were approximately 100 fold more invasive than similar cultures grown without oxygen restriction. This was observed for exponentially growing bacteria, as well as for stationary-phase cultures.

Oral dosage of guinea pigs with *Listeria *resulted in a significantly higher prevalence (p < 0.05) of these bacteria in jejunum, liver and spleen four and seven days after challenge, when the bacterial cultures had been grown under oxygen-restricted conditions prior to dosage. Additionally, a 10–100 fold higher concentration of *Listeria *in fecal samples was observed after dosage with oxygen-restricted bacteria. These differences were seen after challenge with single *Listeria *cultures, as well as with a mixture of two cultures grown with and without oxygen restriction.

**Conclusion:**

Our results show for the first time that the environmental conditions to which *L. monocytogenes *is exposed prior to ingestion are decisive for its *in vivo *infective potential in the gastrointestinal tract after passage of the gastric barrier. This is highly relevant for safety assessment of this organism in food.

## Background

During the last two decades, *Listeria monocytogenes *has been implicated in several food borne outbreaks and sporadic cases of disease [1,2]. Foods implicated in foodborne listeriosis are generally highly processed foods with prolonged shelflives, supportive of growth of the organism [3]. Several attempts have been made to establish quantitative microbiological criteria for the presence of *L. monocytogenes *in foods [4,5], by use of dose-response predictions. However, there are indications that the number of bacteria ingested is not the only important determinant for the development of illness. For many enteric pathogens also including *Listeria*, it is well known that given environmental conditions induce the expression of identified virulence genes and/or contribute to their invasive potential in *in vitro *models [6-15]. However, to our knowledge, no reports describe a direct effect of such conditions on the virulence phenotype of these bacteria *in vivo*. It has thus not been shown whether an induced expression of virulence factors in intestinal pathogens *at the time of ingestion *affects their virulence *in vivo *after passage of the gastric barrier.

The objective of the present study was to investigate whether the physiological state of *L. monocytogenes *prior to ingestion (i.e. determined by the food environment), here exemplified by oxygen availability, could influence its ability to cause infection.

Recent reports point out that mice and rats are not suitable as animal models for human listerial infectivity, since the bacterium does not interact with the epithelial receptor of these animals [16,17]. A guinea pig model [BB Roldgaard, JB Andersen, TR Licht and BB Christensen, submitted] was therefore used for *in vivo *studies of infectivity in the present study, while Caco-2 cells were applied to assess the infective potential *in vitro*. In any animal model, variation between individuals is unavoidable. In order to circumvent this variation in the comparative study, challenge of the animals with a mixture of *L. monocytogenes *ScottA cells cultivated under conditions of different oxygen availability, was included in the investigation. For this purpose, a recently developed fluorescence labeling system [18] was applied, making it possible to distinguish between otherwise isogenic *Listeria *cells originating from cultures grown with and without oxygen-restriction.

The obtained results reveal that oxygen-restriction clearly increases the infective potential of *L. monocytogenes in vitro *and *in vivo*.

## Results

### Invasiveness of oxygen-restricted and un-restricted *L. monocytogenes *in Caco-2 cells

**Figure 1:**
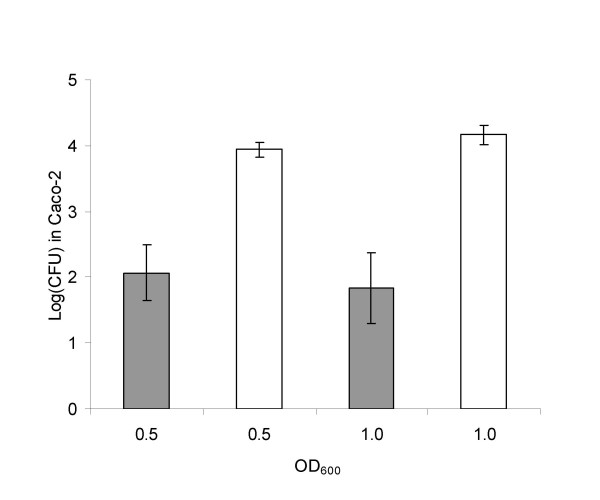
***In vitro *infectivity**. Numbers of invaded un-restricted (grey) and oxygen-restricted (white) *L. monocytogenes*-CFP per well of Caco-2 cells grown to selected densities. Counts were normalized to a concentration of 10^7 ^*L. monocytogenes *per ml in the bacterial cultures prior to infection. Each bar represents an average of three different experiments. Error bars designate standard deviations. OD600 refers to optical density at 600 nm.

Caco-2 cell assays revealed that cultures of *L. monocytogenes*-CFP, grown to selected densities under oxygen-restricted conditions were approximately 100 fold more invasive than corresponding cultures grown without oxygen restriction (Figure 1). Similar results were obtained with *L. monocytogenes*-YFP, as well as with stationary-phase overnight cultures grown with and without oxygen restriction (data not shown).

### Infection of guinea pigs with monocultures of oxygen- restricted and un-restricted *L. monocytogenes*

Exponentially growing cultures (OD_600 _= 1.0) were used for oral dosage of guinea pigs. *L. monocytogenes*-CFP was recovered from the liver and jejunum of half of the 12 animals dosed twice with the un-restricted bacterial cultures, and was found in the spleen of a single animal. The occurrence of *Listeria *in organs of animals dosed with oxygen-restricted bacteria was significantly higher (p < 0.05); the pathogen was recovered from jejunum of all of the 12 animals, and was found in the liver and spleen of ten and seven of the guinea pigs, respectively. The average concentrations of *Listeria *found in positively infected organs were not different in the two groups (Table 1).

**Table 1:** *L. monocytogenes *monoculture infections in guinea pigs

	Un-restricted				Oxygen-restricted			
Days post first dosage	4	7	Total	Mean log(CFU/g)	4	7	Total	Mean log(CFU/g)

Liver	3/6	3/6	6/12 (50%)	2.2 ± 0.34	6/6	4/6	10/12 (83%)	3.0 ± 0.88
Spleen	0/6	1/6	1/12 (8%)	2.8	4/6	3/6	7/12 (58%)	2.6 ± 0.67
Jejunum	2/6	4/6	6/12 (50%)	2.1 ± 0.47	6/6	6/6	12/12 (100%)	3.7 ± 0.70

Numbers of guinea pigs where *L. monocytogenes*-CFP was recovered from liver and spleen after oral dosage with un-restricted or oxygen-restricted *L. monocytogenes*-CFP, respectively. Mean log (CFU/g) designate the mean levels found in animals positive for *Listeria *in the given organs, followed by standard deviations.

**Figure 2:**
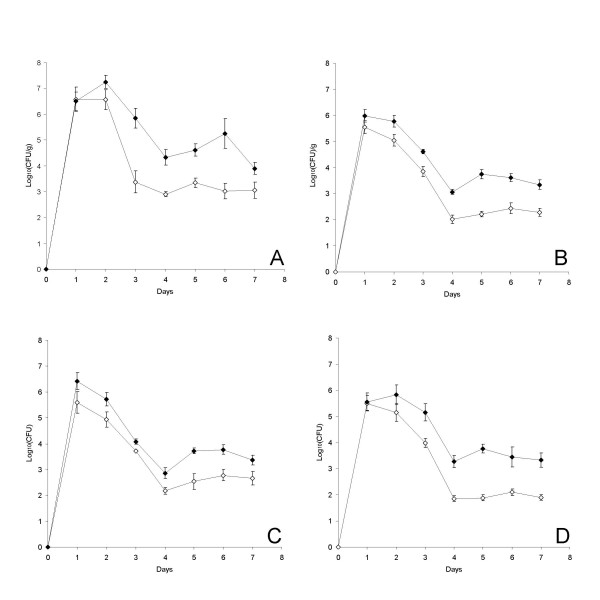
**Fecal densities**. Concentration of *L. monocytogenes *in fecal samples of animals dosed with un-restricted (open symbols) and/or oxygen-restricted (closed symbols), fluorescence-labeled bacteria. (A): Animals dosed with monocultures of CFP-labeled bacteria. (B): Integrated presentation of data from C and D. (C): Animals dosed with a mixture of CFP-labeled, un-restricted *Listeria *and YFP-labeled, oxygen-restricted *Listeria*. (D): Animals were dosed with a mixture of YFP-labeled, un-restricted *Listeria and *CFP-labeled, oxygen-restricted *Listeria*. All animals were dosed at Day 0 and again at Day 1. Each data point in panel A and B represents the average of samples from 12 animals, while each data point in panel C and D represents the average of samples from 6 animals. Error bars designate standard errors of the means.

Throughout the experiment, fecal counts of *Listeria *remained significantly higher in the animals dosed with oxygen-restricted *L. monocytogenes*-CFP than in those dosed with the un-restricted, identical strain. The concentration of *Listeria *in feces reached a level of 10^6^–10^7 ^per gram on the days immediately after dosage, which was carried out on Day 0 and Day 1 of the experiment. Hereafter, the concentration in animals dosed with *L. monocytogenes *grown under un-restricted conditions dropped and remained at a level of approximately 10^3^, while the fecal *Listeria *concentration in animals dosed with oxygen-restricted cells stayed between 1 and 3 logs higher (Figure 2A).

### Competitive infection of guinea pigs with oxygen-restricted and un-restricted *L. monocytogenes*

Oral dosage of 24 guinea-pigs with a mixture of oxygen-restricted and un-restricted, exponentially growing *L. monocytogenes *revealed that also in a mixed challenge experiment, the occurrence of the oxygen-restricted strain in internal organs was significantly higher (p < 0.05) than observed for the un-restricted strain. *Listeria*, which had been grown under un-restricted conditions, was detected in the liver and jejunum of 2 animals, and in the spleen of 4 animals, while *Listeria *grown under oxygen-restricted conditions prior to dosage was recovered from liver, spleen and jejunum of 18, 12, and 14 animals, respectively. It had no influence on the infectivity (p = 0.165) whether the *L. monocytogenes *cells were labeled with CFP or YFP. The average concentrations of *Listeria *found in positively infected organs were similar for oxygen-restricted and un-restricted bacteria (Table 2).

**Table 2:** *L. monocytogenes *mixed culture infections in guinea pigs

	Un-restricted				Oxygen-restricted			
Fluorescence	CFP	YFP	Total	Mean log(CFU/g)	CFP	YFP	Total	Mean log(CFU/g)

Liver	1/12	1/12	2/24 (8%)	1.5 ± 0.21	10/12	8/12	18/24 (75%)	1.9 ± 0.32
Spleen	3/12	1/12	4/24 (17%)	1.9 ± 0.76	7/12	5/12	12/24 (50%)	2.1 ± 0.58
Jejunum	1/12	1/12	2/24 (8%)	2.2 ± 0.21	8/12	6/12	14/24 (58%)	3.1 ± 0.65

Numbers of guinea pigs where *L. monocytogenes *was recovered from liver and spleen after oral dosage with a 1:1 mixture of un-restricted and oxygen-restricted *L. monocytogenes*, carrying either of the two different fluorescent labels CFP and YFP. Samples were taken either four or seven days post first dosage. Mean log (CFU/g) designate the mean levels found in animals positive for *Listeria *in the given organs, followed by standard deviations.

In all animals, fecal concentrations of Listeria grown under oxygen-restricted conditions prior to dosage were higher than concentrations of cells grown without oxygen restriction (Figure 2B). This was observed independently of which fluorescent label was used to identify the bacteria (Figure 2C and 2D). The kinetics of the pathogen occurrence in feces observed in the mixed infection experiment were quite similar to what was observed after dosage with monocultures (Figure 2).

## Discussion

We have shown that oxygen-restriction prior to ingestion significantly (p < 0.05) enhances the *in vivo *infective potential of *L. monocytogenes *ScottA (Table 1 and 2, Figure 2). This has to our knowledge not previously been shown, and signifies that gene expression occurring in *Listeria *before intake influences its infective potential even after passage of the oral/gastric barrier. The trend in the results is that the prevalence of *Listeria *in internal organs increases from Day 4 to Day 7 post challenge with the un-restricted bacteria, while a decrease in prevalence is seen in the same period after dosage with the oxygen-restricted bacteria (Table 1). The effect of oxygen-restriction prior to dosage is thus larger on Day 4 than on Day 7 post dosage, which is not surprising because the expression patterns present in the cultures at the time of ingestion must be expected to approach each other over time, since the cells are all exposed to similar environmental conditions in the gut.

While oxygen restriction clearly affected the number of animals carrying *L. monocytogenes *in their internal organs, the concentration of bacteria present in positively infected organs was not affected (Table 1 and 2). This suggest that oxygen restriction increases the initial translocation of *Listeria *from the gut lumen to internal organs, but does not influence the ability of the bacteria to proliferate inside the investigated organs.

Obviously, an increased ability to survive the gastric barrier will increase the probability of causing an infection [19,20]. It is well known, that genes involved in many kinds of stress-responses are co-regulated, and that exposure to one type of stress therefore improves the ability to survive another type of stressful condition [21,22]. It could thus be speculated, that exposure to oxygen restriction would increase the viability of *L. monocytogenes *under low pH and/or its resistance to gastric enzymes. This is however not the full explanation for our observations, since also the ability of this strain to infect Caco-2 cells *in vitro *is significantly increased by oxygen restriction (Figure 1). In the *in vitro *studies, the effect of oxygen restriction was seen for exponentially growing cultures as well as for cultures in stationary phase, and the induction of the virulent phenotype was thus expected to be independent of the growth stage of the bacterial cells (Figure 1). Still, we chose to use exponentially growing cultures for the animal studies in order to eliminate putative contributions to *in vivo *infectivity from the many genes known to be induced in stationary phase.

We suggest that the observed increased infectivity of *L. monocytogenes *grown under oxygen-restricted conditions can be attributed to an increased expression of the InternalinA (InlA) protein, which is known to be a key factor for virulence of *L. monocytogenes *[23], on the surface of the bacterial cells. In concordance with this hypothesis, recent reports indicate that anaerobic physiology contributes significantly to an enhanced production of InlA in *aro *mutants of *L. monocytogenes *[24,25]. InlA mediates the interaction of *Listeria *cells with specific receptors (E-cadherin) in the human gut [26], and may therefore be important for bacterial attachment to the epithelial wall. We observed that oxygen-restriction significantly increased the prevalence of *L. monocytogenes *in the jejunum of guinea-pigs (Table 1 and 2), suggesting that an increased InlA-expression occurring prior to ingestion caused increased attachment of *L. monocytogenes *to the jejunal mucosa. We speculate that the observed increased translocation to spleen and liver (Tables 1 and 2), as well as the increased invasion of Caco-2 cells (Figure 1) may be attributed to an increased initial InlA-mediated attachment to the epithelial receptors. However, also other genes are reported to be induced under oxygen restricted conditions and to affect attachment of *Listeria *[27].

The percentage of animals in which *Listeria *was recovered from internal organs was significantly lower (p < 0.05) in animals infected with mixed cultures, than in animals infected with monocultures (Tables 1 and 2). The sensitivity of the mixed culture infection approach was thus slightly lower than observed for infection with monocultures, probably because the dosed numbers of cells grown under a given condition in the mixed infections were only half of the corresponding numbers dosed as monocultures. Total numbers of cells in each dosage were similar in the two approaches. In spite of this, the observed difference between oxygen-restricted and un-restricted *Listeria *was more significant in the mixed culture experiment (p_liver _< 0.0001; p_spleen _= 0.0143; p_jejunum _= 0.0002) than in the monoculture infections (p_liver _= 0.0833; p_spleen _= 0.0094; p_jejunum _= 0.0047) due to the higher amount of data resulting from the mixed-infection approach. This shows that the co-infection model, which has the advantage of elimination of variations attributed to individual animals, additionally had a better discriminatory power than a monoculture approach involving the same number of animals.

## Conclusion

Our results are of particular importance for the risk assessment of *Listeria *in food. For the first time, we have shown that the environmental conditions to which a bacterium is exposed before ingestion can be decisive for its infective potential when it reaches the gut. This means that not only the number of *Listeria *present in a given food item, but that also the physiological condition of these bacteria is important for food safety. The *in vitro *and *in vivo *data suggest that an oxygen-restricted *L. monocytogenes *cell represents a significantly higher risk than a cell grown without oxygen restriction. This should be taken into account in future quantitative risk profiling and dose-response models.

## Methods

### Strains and media

The clinical isolate, *Listeria monocytogenes *ScottA, carrying erythromycin resistance, and labeled with either Cyan Fluorescent Protein (CFP) or Yellow Fluorescent Protein (YFP) as previously described [18] was used in all experiments. Bacteria were cultivated on BHI-agar (Oxoid) or in liquid BHI (Oxoid) buffered with 100 mM 3-(N-Morpholino)-propanesulfonic acid (MOPS), pH = 6. When appropriate, Erythromycin (Sigma) was used at a final concentration of 10 ug/mL and Nalidixic acid at a final concentration of 100 ug/mL.

### Preparation of *L. monocytogenes *for infection studies

A fluorescent single colony of *L. monocytogenes *was inoculated into 10 mL MOPS buffered BHI media supplemented with Erythromycin and incubated at 37°C for 8 hours. The resulting cultures, for which the optical densies (OD_600_) ranged between 1,5 and 2, were subsequently diluted between 10^4 ^and 10^5 ^fold into 2 L Bluecap flasks containing 450 mL MOPS buffered BHI medium supplemented with Erythromycin.

To obtain oxygen-restricted cultures of *Listeria monocytogenes *ScottA, atmospheric air above the diluted cultures were exchanged with sterile Nitrogen and the lid of the Bluecap flasks was tightened and sealed prior to incubation. To obtain non-restricted cultures, the Bluecap flasks were incubated with the lid loosely tightened allowing free exchange of atmospheric air. All cultures were incubated at 37°C in a rotary shaker set at 200 rpm and samples for *in vitro *invasion studies and *in vivo *infection studies were taken after approximately 20 hours of incubation, when they reached the optical densities reported below.

Samples for *in vitro *invasion studies were taken at OD_600 _= 0.5 (exponential phase), OD_600 _= 1.0 (exponential phase), and from cultures that had been in stationary phase for approximately 10 hours (referred to as over night cultures). The samples were diluted directly into 37°C MEM medium (Gibco) to a concentration of 10^7 ^bacteria/mL and immediately used for challenge of Caco-2 cells as described below.

Samples for oral challenge of guinea pigs were cultivated until OD_600 _= 1, and 400 mL culture per group of 6 animals was harvested by centrifugation (5 minutes, 7000 g, 25°C). The pellet was resuspended in 4 mL of double cream (38 % fat, pH 7). Aliqouts of 0.5 mL, containing either *Listeria *monocultures, or a mixture of two cultures grown at oxygen restricted and unrestricted conditions, respectively, were subsequently administered to guinea pigs as described below. Samples of the inoculums were diluted and spread onto BHI-agar supplemented with Erythromycin to estimate the numbers of *Listeria *present in the double cream suspensions. Microscopy revealed that the bacteria were present in the hydric phase.

### Caco-2 cell infection experiments

Enterocyte-like Caco-2 cells were cultivated and prepared as previously described [28]. Bacteria were cultivated as described above to the desired optical density and diluted in 37°C MEM medium (Gibco) to a concentration of 10^7 ^bacteria/mL immediately before the invasion assays. One ml of bacterial culture was applied to each well, resulting in a multiplicity of infection of approximately 25 bacteria per Caco-2 cell. Following 1 hour of invasion and 2 hours of gentamycin treatment to kill extracellularly located bacteria, Caco-2 cells were lysed and the numbers of *Listeria *present in each well was estimated as described in the section 'Enumeration of *L. monocytogenes *in samples'. Sampling was done in triplicate, and the experiments were performed twice.

### Animal experiments

Male and female Hartley guinea pigs (Charles River Laboratories; Germany) with a weight of 275 g (± 10 g) were used. After seven days of acclimatization in pens (custom made, 90 × 130 × 61 cm), animals were randomized and housed individually in Polycarbonate cages, Eurostandard Type III H (425 × 266 × 185 mm) with Tapvei bedding (peeled Aspen hardwood, Tapvei Kaavi, Finland) in negatively pressurized isolators. Fecal samples from the guinea pigs were tested by plating on Palcam agar (Oxoid) to verify the absence of *Listeria *prior to dosage.

A total of 48 guinea pigs were dosed with *L. monocytogenes *ScottA. Two groups of 12 animals were dosed with monocultures of CFP-labelled bacteria, cultivated either with or without oxygen-restriction. Two other groups of 12 animals were dosed with a 1:1 mixture of oxygen-restricted and un-restricted bacteria carrying the two different fluorescent labels CFP and YFP. In one of these groups, it were the oxygen-restricted *Listeria*, which carried the CFP label, while in the other group the labels were reversed so that the unrestricted bacteria were labelled with CFP.

All animals were dosed with 0.5 ml double cream containing 38 % milk fat and approximately 5 × 10^10 ^*L. monocytogenes *at Day 0 and again at Day 1. The number of cells in the inoculum was approximately the same in the mono- and mixed cultures.

Dosage was done directly in the oral cavity, between the incisors and the molars. Following dosage the animals had access to food and water *ad libitum *throughout the duration of the study. Fresh fecal samples were collected every day, avoiding bedding and traces of urin from the cages. Six animals from each of the groups were euthanized on Days 4 and 7, respectively, for investigation of intestinal segments, liver and spleen. Samples of jejunal content from the middle part of the small intestine were squeezed out with tweezers, and samples from spleen and liver of approximately 0.6 g were homogenized prior to investigation as described below.

### Ethical aspects

Animal experiments were carried out under the supervision of the Danish National Agency for Protection of Experimental Animals

The recently developed guinea-pig model [BB Roldgaard, JB Andersen, TR Licht and BB Christensen, submitted] is much less stressful to the animals than previously published models [17], since it involves no intubation, injection or anesthetizing. Additionally, the approach of treating each animal with a mixture of cultures reduces the number animals needed.

### Estimation of *L. monocytogenes *in samples

Samples of lysed Caco-2 cells and homogenized organs were cultivated on BHI-agar supplemented with Erythromycin. Samples from faeces and jejunum were cultivated on BHI-agar supplemented with Erythromycin and Nalidixic acid. After 48–72 hours of incubation at 37°C, BHI plates were placed on a UV table (excitation at 302 nm), and fluorescent colonies (either CFP or YFP) were enumerated.

### Statistics

Statistical analysis was performed using the software package JMP (SAS institute, Copenhagen, Denmark). Pearsons Chi^2 ^test was used to compare the numbers of infected animals after dosage with either oxygen-restricted or un-restricted *L. monocytogenes*. The same test was used to compare the results obtained with either mono infection or mixed infection, and with either YFP or CFP labelling. A significance level of 0.05 was used in all cases.

## Authors' contributions

JBA carried out the sample preparation and the in vitro invasion assays, while BBR was responsible for the animal experiments. BBC and TRL conceived the study and participated in its design and coordination. TRL drafted the manuscript. All authors read and approved the final manuscript.
